# Dataset of metal(loid) concentrations recorded in the tissues of two fish species from Flag Boshielo Dam, South Africa

**DOI:** 10.1016/j.dib.2020.106396

**Published:** 2020-10-09

**Authors:** Jeffrey Lebepe, Paul J. Oberholster, Wilmien J Luus-Powell

**Affiliations:** a School of Life Sciences, University of KwaZulu-Natal, Durban 4000, South Africa; b Centre for Environmental Management, University of the Free State, Bloemfontein 9300, South Africa; c Department of Biodiversity, DST-NRF SARCHI (Ecosystem Health), University of Limpopo, Sovenga 0727, South Africa

**Keywords:** Olifants River, Metal(loid)s, Flag Boshielo Dam, *Oreochromis mossambicus*, *Labeo rosae*, Cadmium, manganese, Lead

## Abstract

Metal(loid) pollution in aquatic ecosystems has become a cause for concern, particularly in areas where communities depend on services from these systems for their livelihood. This dataset presents the metal(loi) concentrations recorded in the water column, bottom sediment, and tissues of *Oreochromis mossambicus* and *Labeo rosae* from Flag Boshielo Dam, an impoundment in one of the most polluted river systems in Southern Africa, the Olifants River. The concentrations of metal(loid)s were measured using inductively coupled plasma-optical emission spectrophotometry (ICP-OES; Perkin Elmer, Optima 2100DV). The data generated attest that in aquatic ecosystems, metal(loid)s do not remain in suspension in the water column, but sink down to the bottom sediment where they accumulate or get taken up by receptor organisms such as fish. It further confirm that there is a clear separation on the extent to which metal(loid)s are accumulating in different tissues and liver mostly accumulate higher concentration followed by gills and muscle, respectively. These data can be useful to guide future studies aiming to understand the dynamics, pathways and fate of metal(loid)s in relation to water, sediment and fish tissues. These data can also be used for decision making in relation to the establishment of freshwater fisheries in dams receiving metal(loid)s from different land use activities.

## Specifications Table

**Table:** 

Subject	Environmental Science (Pollution)
Specific subject area	Metal(loid) accumulation in freshwater fish
Type of data	Tables Figures
How data were acquired	The data was acquired through field sampling and laboratory analysis using inductively coupled plasma-optical emission spectrometry (ICP-OES) (Perkin Elmer, Optima 2100 DV).
Data format	Raw Filtered Analyzed
Parameters for data collection	The data was collected during the winter and summer seasons to cover seasonal variation of flow levels and climate. Different fish sizes were collected to cover a wide variety of length.
Description of data collection	Fish sampling was undertaken during winter and summer seasons with the aid of gill nets in 2014. Collected fish were sacrificed by severing the spinal and the weight and lengths were recorded before opening them ventrally using dissecting scissors. Fish muscle, gills and liver were harvested and wrapped with aluminium foil, kept in dry ice and later transferred to −20 °C freezer. Samples were taken to South African National Accreditation System (SANAS) accredited laboratory (ISO/IEC 17025:2005) for metal(loid) analyses. In the laboratory, tissue samples were analysed in batches with blanks using inductively coupled plasma-optical emission spectrophotometry (ICP-OES; Perkin Elmer, Optima 2100DV).
Data source location	Flag Boshielo Dam: 24°46ʹ S, 29°25ʹ E Province: Limpopo Country: South Africa
Data accessibility	With the article and attached as a supplementary material named Appendix A.

## Value of the Data

• This data can be used as a guide when planning studies to investigate pathways and distribution of these metal(loid)s in contaminated environment.
• The data is of primary importance to ecotoxicology research communities as it provides an insight on the dynamics, pathways and fate of these metal(loid)s in relation to water column, bottom sediment and fish tissues.
• This data may be used to explore possible trends to be expected with respect to metal(loid) concentrations in these three media in a contaminated environment.
• These data gives an insight on what to expect in metal(loid)s contaminated waterbodies, hence, help with decision making when considering inland fisheries in a possibly contaminated water bodies.

## 1 Data Description

This dataset present the dynamics, pathways and fate of metal(loid)s in an aquatic ecosystem, Flag Boshielo Dam. Table 1 present the levels of physico-chemical parameters reported at Flag Boshielo Dam during winter and summer in 2014. All metal(loid)s of concern were measured in the water column, nevertheless, only few were detected, hence the table was filtered and undetected metal(loid)s were not included. The concentration of aluminium (Al), arsenic (As), cadmium (Cd), chromium (Cr), copper (Cu), manganese (Mn), lead (Pb), antimony (Sb) and zinc (Zn) were not detected in the water column. Table 2 present concentrations of metal(oids) recorded in the bottom sediment measured in mg/kg dry weight. Moreover, As and Sb were below detection limit in the bottom sediment across all sites during both seasons, hence, they were not included on the table.

**Table 1:** Levels of physico-chemical parameters recorded in the water at Flag Boshielo Dam during winter and summer 2014.

	Summer			Winter		
Constituents	Inflow	Middle	Dam wall	Inflow	Middle	Dam wall
Temperature (°C)	25.90	25.20	26.20	19.92	17.89	17.82
DO (mg L^−1^)	10.33	10.51	10.26	9.54	9.25	8.55
pH	7.80	8.70	8.40	8.60	9.00	9.20
TDS (mg L^−1^)	306.21	312.00	334.80	375.70	312.00	302.90
Conductivity (mS/m)	47.10	48.00	51.50	57.80	48.00	46.66
Fe (mg L^−1^)	0.01	0.01	0.01	nd	nd	nd
Se (mg L^−1^)	nd	nd	nd	0.02	0.02	0.02
Sr (mg L^−1^)	0.15	0.16	0.16	0.16	0.16	0.18

nd: below detection limit.

**Table 2:** Metal(loid) concentrations (mg/kg dry weight) recorded in the bottom sediment at Flag Boshielo Dam during summer and winter 2014.

	Summer			Winter		
Metal(loid)s	Inflow	Middle	Dam wall	Inflow	Middle	Dam wall
Al	18000.00	66000.00	17200.00	38400.00	12800.00	9200.00
As	nd	11.06	nd	9.80	nd	nd
Cd	nd	nd	nd	nd	nd	nd
Cr	40.050	109.56	17.140	68.270	32.870	8.24
Cu	11.670	42.650	10.310	25.130	9.1100	5.04
Fe	44800.00	68000.00	16800.00	42800.00	37200.00	15600.00
Mn	800.80	1343.60	317.20	932.80	573.20	214.40
Pb	11.36	35.51	10.10	23.08	7.67	10.25
Sb	nd	nd	nd	nd	nd	nd
Se	nd	nd	20.530	19.210	nd	nd
Sr	19.91	30.09	5.74	26.67	18.77	5.52
Zn	54.80	128.80	115.00	94.80	47.60	23.20

**Fig. 1:**
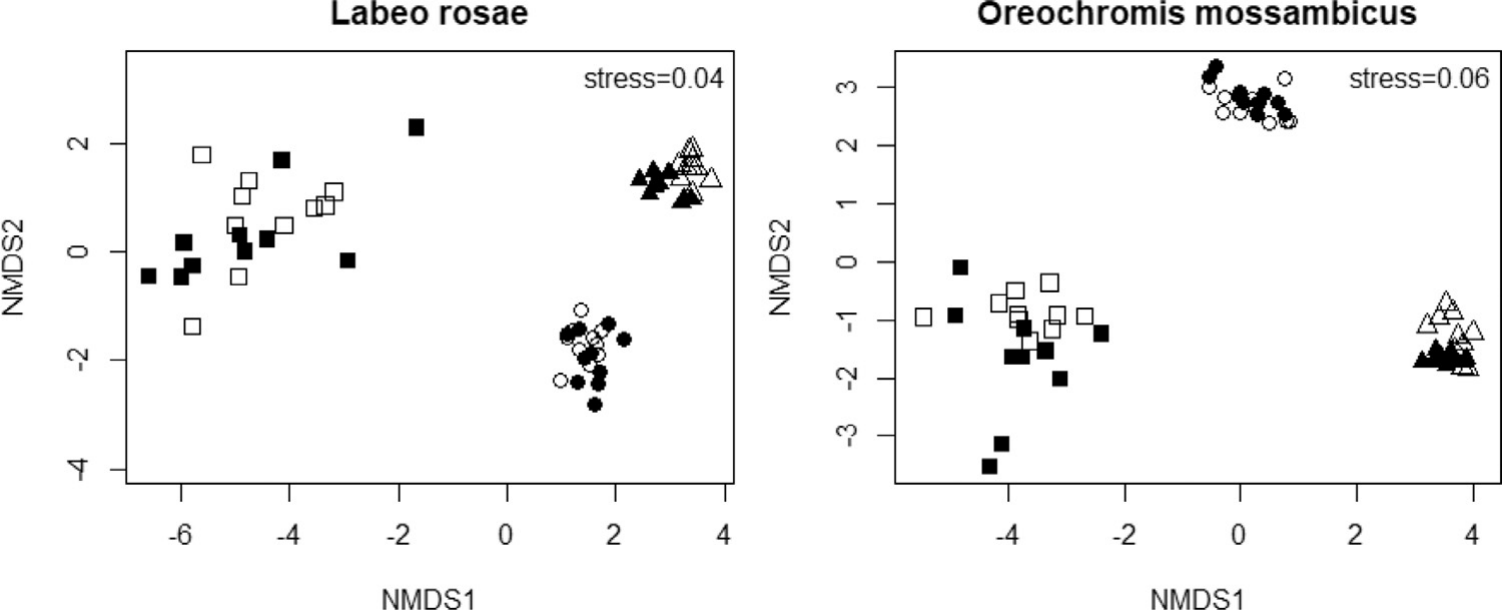
Non-metric multidimensional scaling ordination for metal(loid) concentration recorded for both species in the liver (square), gill (circle) and muscle (triangle) at Flag Boshielo Dam during summer (unfilled) and winter (filled) in 2014.

**Fig. 2:**
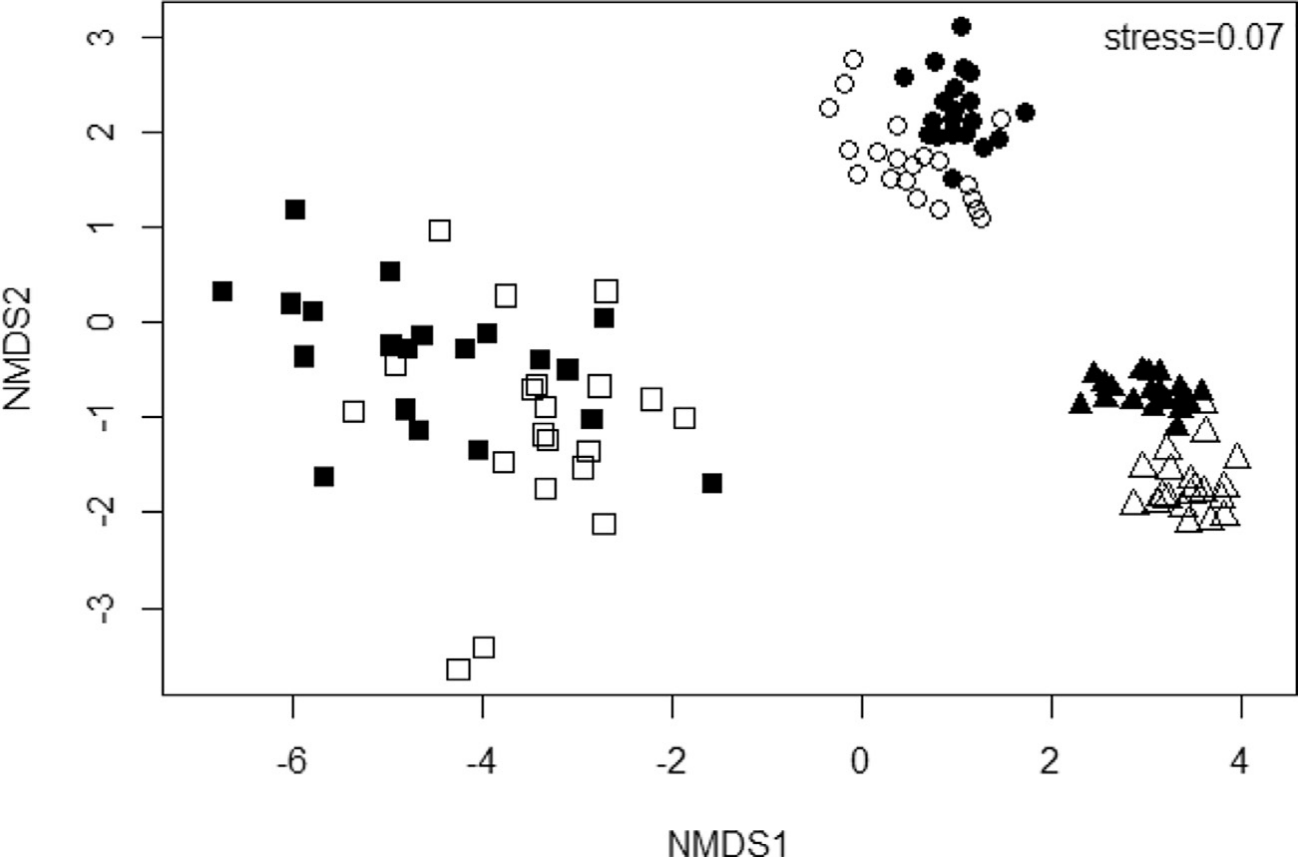
Non-metric multidimensional scaling ordination comparing metal(loid) concentration recorded in the liver (square), gill (circle) and muscle (triangle) of *Labeo rosae* (filled) and *Oreochromis mossambicus* (unfilled) from Flag Boshielo Dam during 2014 surveys.

Fish morphometry, weight and total length are presented in a supplementary file, Appendix A, in a spreadsheet named fish morphometrics. Appendix A also present the concentrations of metal(loid)s recorded in the liver, gill and muscle of *Labeo rosae* and *Oreochromis mossambicus* during winter and summer. Fig. 1 present non-metric multidimensional scaling ordination for metal(loid) concentrations recorded in three tissues for fish tissues whereas Fig. 2 brought both species together to check if there was separation for each organ between the two species. The dispersion of metal(loid) concentrations in different tissues for both species was also projected by these two figures (Figs 1 and 2).

## 2 Experimental Design, Materials and Methods

### 2.1 Sampling area

**Fig. 3:**
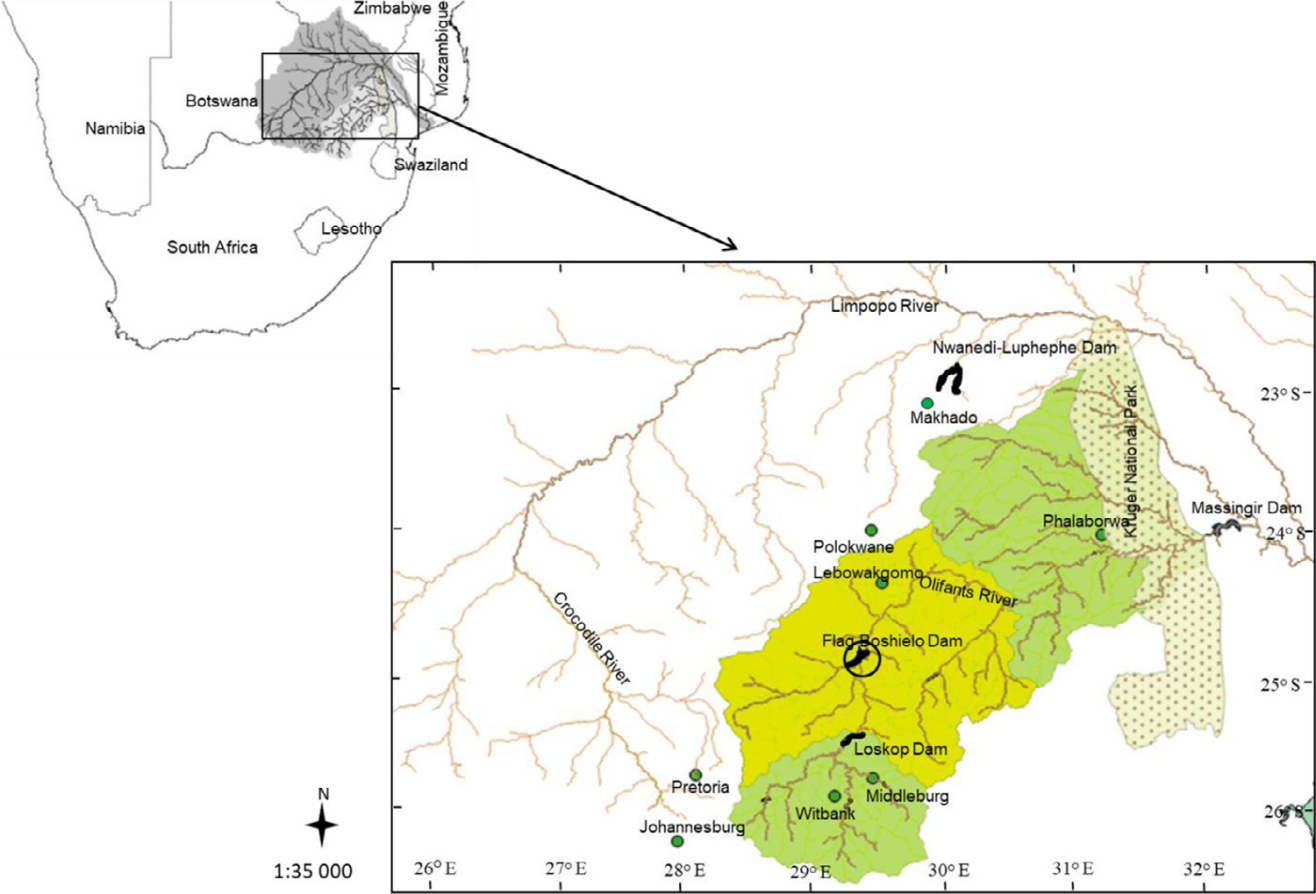
The Olifants River catchment with Flag Boshielo Dam encircled.

The data was collected at Flag Boshielo Dam (24°46ʹ S, 29°25ʹ E), an impoundment in one of the most polluted river system in South Africa, the Olifants River system [1]. The dam is located approximately 25 km north-east of Marble Hall town (Fig. 3) and it was built to supply water for irrigation, domestic and industrial and for recreational purposes [2]. This dam is among those recommended by South African government for inland fisheries.

### 2.2 Surface water and bottom sediment sampling

To get a clear representation of the whole dam, water and sediment sampling was carried out at the inflow, middle and dam wall. Physical parameters, namely, temperature, dissolved oxygen, pH, total dissolved solids and conductivity were measured *in situ* with the aid of a handheld multi-parameter YSI (Model 556) at each site. For chemical analysis, water samples were collected using acid treated water bottles and kept in the fridge (−4°) and later analyzed for metal(loid)s. For sediment analysis, a composite of three grab sample was collected at the inflow, middle and dam wall. Prior metal(loid)s analysis, sediment samples were stored in acid treated water bottles and frozen.

### 2.3 Fish sampling and processing

The collection of fish was undertaken at Flag Boshielo Dam during winter (low flow) and summer (high flow) seasons in 2014 with the aid of gill nets with mesh sizes ranging from 50 to 150 mm stretched and 3m drop. Ten fish specimens were collected for each species during each survey. Fish are easy to identify using morphological characters, therefore, identification was carried out based on morphological characters found in the guide for Southern Africa freshwater fish [3]. Fish were sacrificed by severing the spinal and opened ventrally using dissecting scissors. Prior fish dissection, weight (g) and lengths (cm) were recorded. The muscle, gills and liver were harvested and wrapped with aluminium foil, kept in dry ice, and later transferred to -20 °C freezer. Ethical clearance was approved by the University of Pretoria Animal Use and Care ethical committee (reference number T001-12).

### 2.4 Metal(loid) analysis

Water and sediment samples were analysed following Bervoets and Blust [4] protocol. For water analysis, a 0.45 µm filter paper was used for filtration and the water was acidified to a pH of 2 with 1 M ultrapure nitric acid (Merck, Germany). For sediment analysis, extraction was done using 0.1 M NH_2_OH.HCl in 0.01 HNO_3_ at room temperature for 30 h with a 15:1 solution. The solution was filtered through 0.45 μm filter paper under vacuum, stored in a presterilised acid-washed volumetric flask rinsed with deionised water. For fish samples, tissue were thawed and rinsed with deionised water. Approximately 5g of wet tissue was dried in an oven at 60 °C for 48h. The dried tissues was also weighed to determine the moisture content of the samples. Tissue digestion was performed using the protocol adapted from Islam et al. [5] and Pollet and Bendell-Young [6]. Dried tissues were digested using suprapur grade 7 mL 69% nitric acid (HNO_3_) and 1 mL 30% hydrogen peroxide (H_2_O_2_) (Merck, Germany) in a microwave digestive system. The digestion steps included keeping the temperature at 180 °C and power at 80% for 15 min, then temperature at 190 °C and power at 90% for 15 min and for cooling down the temperature decreased to 100 °C with power of 40% for 10 min. After 10 min in 100 °C solution was kept in cold water. Once cooled, each solution was filtered through 0.45 μm filter paper under vacuum and stored in a presterilised acid-washed volumetric flask rinsed with deionised water. Water, sediment and fish solutions were made to the mark of 50 mL by adding deionised water and stored at 4 °C prior to the determination of metal(loid) concentration. Metal(loid)s analysis was performed in batches including blanks as controls using inductively coupled plasma-optical emission spectrophotometry (ICP-OES; Perkin Elmer, Optima 2100DV) with the detection limit of 0.01 mg/L. All analysis were done in duplicate. Validation was carried out using DORM-4 certified reference materials for fish supplied by Canadian National Research Council (CNRC), and certified and the recoveries ranging from 93.04 to 115.53%.

## Declaration of Competing Interest

The authors declare that they have no conflict of interest.

## Appendix Supplementary materials

Image, application 1

## Footnotes

Supplementary material associated with this article can be found in the online version at doi:10.1016/j.dib.2020.106396.
